# Maternal fever during pregnancy and offspring attention deficit hyperactivity disorder

**DOI:** 10.1038/s41598-019-45920-7

**Published:** 2019-07-02

**Authors:** Kristin Gustavson, Helga Ask, Eivind Ystrom, Camilla Stoltenberg, W. Ian Lipkin, Pål Surén, Siri E. Håberg, Per Magnus, Gun Peggy Knudsen, Espen Eilertsen, Michaeline Bresnahan, Heidi Aase, Siri Mjaaland, Ezra S. Susser, Mady Hornig, Ted Reichborn-Kjennerud

**Affiliations:** 1 Norwegian Institute of Public Health, Oslo, Norway; 2 University of Oslo, Oslo, Norway; 3 University of Bergen, Bergen, Norway; 4 Department of Epidemiology, Columbia University Mailman School of Public Health, New York, NY USA; 5 Center for Infection and Immunity, Columbia University Mailman School of Public Health, New York, NY USA; 6 Departments of Neurology and Pathology, Mailman School of Public Health, New York, NY USA; 7 College of Physicians and Surgeons, Columbia University, New York, NY USA; 8 New York State Psychiatric Institute, New York, NY USA; 9 Centre for Fertility and Health, Norwegian Institute of Public Health, Oslo, Norway

**Keywords:** Epidemiology, Paediatric research, Risk factors

## Abstract

Maternal fever during pregnancy is associated with several adverse child outcomes. We investigated associations between maternal fever and ADHD among offspring, as well as the sub-dimensions of ADHD - inattention and hyperactivity/impulsivity. Data came from the Norwegian Mother and Child Cohort Study, including more than 114,000 children. Information about children’s ADHD diagnoses was obtained from the Norwegian Patient Register. Mothers reported on inattention and hyperactivity/impulsivity symptoms in questionnaires at 8 years. Logistic regression analysis showed that children exposed to maternal fever in the first trimester received an ADHD diagnosis more often than unexposed children (Odds Ratio (OR) = 1.31, 95% confidence interval (CI) = 1.06–1.61). For children exposed twice or more in the first trimester, the OR was 2.64 (CI = 1.36–5.14). Linear regression analysis showed elevated inattention symptoms among children exposed to fever in the first (Cohen’s d = 0.09, CI = 0.03–0.15) and second (Cohen’s d = 0.05, CI = 0.01–0.09) trimester. Results were similar whether the mother had taken acetaminophen for their fever or not. Hyperactivity/impulsivity symptoms were not related to maternal fever. The results indicate that maternal fever in early pregnancy may be a risk factor for ADHD, and particularly for inattention problems. This risk is neither mitigated nor inflated by use of acetaminophen.

## Introduction

ADHD is the most common psychiatric diagnosis among children, and prenatal environmental exposures have been proposed as risk factors^1,2^. Maternal fever during pregnancy is associated with several adverse child outcomes, such as neural tube defects, brain damage, autism spectrum disorders, lack of task persistence, and academic outcomes^3–8^, but the association with ADHD is uncertain.

Adverse outcomes may be due to infections that culminate in fever, or to hyperthermia itself^5^. Studies of humans and animals exposed to hyperthermia unrelated to infections (e.g. hot tubs and unusually hot weather) indicate that elevated temperatures may have teratogenic effects^5,9,10^. Cells in the central nervous system seem to be particularly vulnerable to hyperthermia^9,11^. Disturbances in brain growth and subsequent neurobehavioral problems may thus occur in the offspring^12^. Teratogens often have different effects depending on timing^13^. In the case of fever, first-trimester exposure has been linked to brain damage and neural tube defects^14^, and second-trimester exposure to autism spectrum disorder^6^ and lack of task persistence^3^.

It is not clear whether maternal fever during pregnancy is associated with ADHD, and if timing of exposure is of importance. In a recent study based on a Danish birth cohort, an association was found between child ADHD diagnosis and maternal fever during gestational weeks 9 to 12, but not during the full pregnancy period^15^. We also lack knowledge about the degree to which maternal fever in pregnancy is equally associated with each of the two sub-dimensions of ADHD – inattention and hyperactivity/impulsivity. These sub-dimensions may have different risk factors^16–18^. Hence, a better understanding of the development of ADHD requires examination of risk factors for the diagnosis as well as for each of the sub-dimensions.

Exposure to multiple fever episodes during pregnancy may be associated with particularly high risk of ADHD. Increased risk with higher number of fever episodes has been shown for autism spectrum disorders^6^, but this has not been examined for ADHD and its sub-dimensions.

Use of the antipyretic and pain reliever acetaminophen during pregnancy has been associated with adverse neurodevelopmental outcomes^19^, including ADHD^20,21^. An observed association between maternal fever in pregnancy and child ADHD could thus potentially be due to acetaminophen use for fever. It is therefore important to account for acetaminophen when examining the association between maternal fever and child ADHD.

The aims of the current study were to examine associations between: i) maternal fever during pregnancy and ADHD diagnosis in offspring, ii) maternal fever in different trimesters and child ADHD diagnosis, iii) maternal fever during pregnancy and maternal reports of symptoms of inattention and hyperactivity/impulsivity when children were 8 years old, iv) multiple fever episodes during pregnancy and child ADHD (diagnosis and symptoms), and v) maternal fever during pregnancy and ADHD (diagnosis and symptoms) in children of mothers who did versus did not take acetaminophen for their fever.

## Methods

### Study population

We used data from the Norwegian Mother and Child Cohort Study (MoBa)^22,23^. Participants were recruited between 1999 and 2008 from hospitals all over Norway. Women received information about the study, a consent form, and the first questionnaires with their postal invitation to routine ultrasound examination around gestational week 17. There were no exclusion criteria, but questionnaires were in Norwegian, so the ability to read Norwegian was a restriction^22,23^.

MoBa follows more than 114,000 children from almost 113,000 pregnancies (40.6% of the invited), 95,000 mothers, and 75,000 fathers. Participants received three questionnaires during pregnancy (gestational weeks 17, 22, and 30) and several questionnaires after the child was born (age 6 months, 18 months, 3 years, 5 years, 7 years, and 8 years).

The current study used data from mothers’ questionnaires in gestational weeks 17 (n = 99,947 children) and 30 (n = 91,371), as well as 6 months (n = 85,546), 3 years (n = 56,526), and 8 years (n = 41,543) after birth. All available information from participants with missing data was included in analyses. This is generally recommended to reduce risk of bias due to non-response^24^. One exception was that only women responding to all fever-related questionnaires were included when examining associations between fever in different trimesters and child ADHD (diagnosis and mother-reported symptoms). This was done to ensure that the exact same sample was used for all trimesters, so that we did not detect differences between trimesters that were in fact due to slightly different samples in the three questionnaires.

Only children from singleton births were included, in line with previous studies on prenatal risk factors for ADHD^15,25,26^. Children born in the year 2009 or later (n = 3,153) had not received the 8-year questionnaire by the end of the current study period in 2016, and were excluded from analyses with mother-reported symptoms at 8 years as outcome.

**Figure 1:**
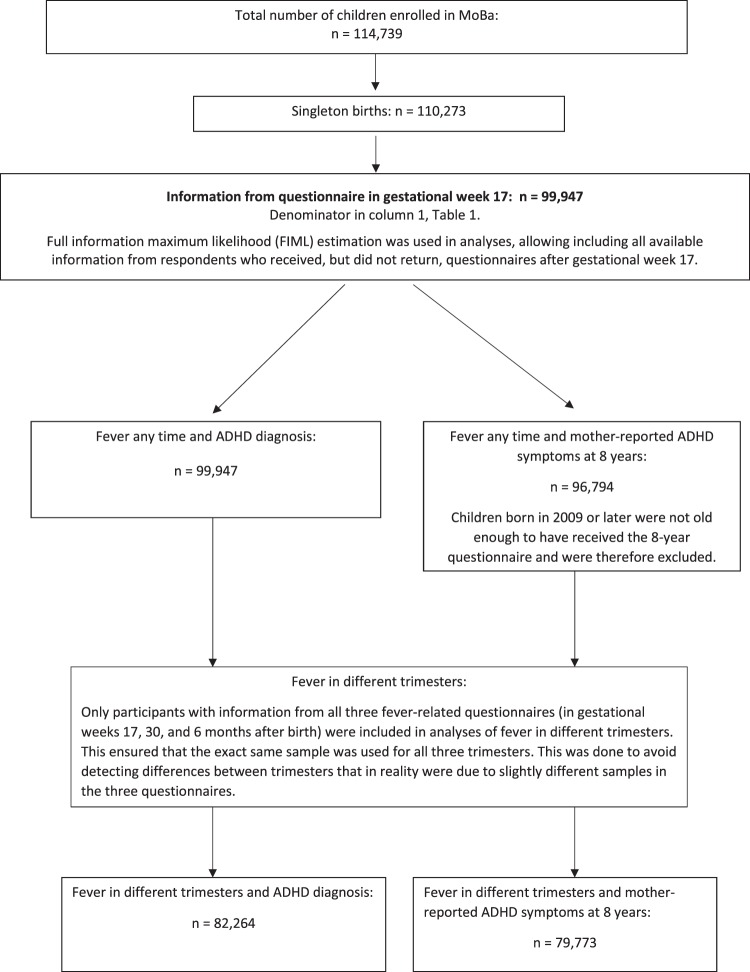
Flow chart of numbers of participants in the analyses.

For details of eligibility, see flow-chart in Fig. 1.

MoBa has a license from the Norwegian Data Inspectorate. Participants have provided written informed consent. The Regional Committee for Medical Research Ethics approved MoBa and the current study. All methods were performed in accordance with relevant guidelines and regulations.

### Exposures

Mothers reported fever episodes during pregnancy in questionnaires at gestational weeks 17 and 30, and at 6 months after pregnancy. In week 17, women were asked if they had fever above 38.5 degrees Celsius (101.3 degrees Fahrenheit) during each of the following four time periods: gestational weeks 0–4, 5–8, 9–12, and 13 or later. In week 30, women were asked if they had fever once, twice, three times, or more than three times from gestational week 13 onwards. For the first three fever episodes, they were asked to specify time period (i.e. gestational weeks 13–16, 17–20, 21–24, 25–28 and 29 or later), and the highest temperature of each episode. We included episodes with a temperature of 38.5 degrees Celsius or more as fever episodes, because women had only been asked about fever of this temperature or higher in the first questionnaire. Mothers reported fever in the last part of pregnancy in the questionnaire administered 6 months after delivery. They were then asked if they had fever in the last part of pregnancy, since completion of the previous questionnaire (yes/no). Fever episodes in gestational weeks 0–12 were defined as fever in first trimester, fever in weeks 13–28 as fever in second trimester, and in weeks 29 and after as fever in third trimester.

Number of fever episodes were counted in the first and second trimester. Fever episodes reported in two different questionnaires for the same period were counted only once. For each fever episode, women were asked what medication they had taken. Use of acetaminophen for each fever episode was coded yes/no, comprising any dose or duration.

### Outcomes

From 2008, all government-funded in-patient and out-patient clinics in Norway report diagnoses to the Norwegian Patient Register (NPR). Children registered with Hyperkinetic disorder (F90) according to the 10^th^ revision of the International Classification of Diseases (ICD-10)^27^ between 2008 and 2016 were defined as ADHD cases in the current study.

When the children in MoBa were 8 years, mothers responded to the ADHD Rating Scale^28^, which includes 18 items regarding ADHD from the Diagnostic and Statistical Manual of Diseases - 4^th^ revision (DSM-IV)^29^. Nine items were related to hyperactivity/impulsivity, and nine to inattention. Mothers rated each statement on a scale from 1 (“never/rarely”) to 4 (“very often”). Examples of items were: “Fails to give close attention to details or makes careless mistakes in schoolwork”, “Is easily distracted”, and “Has difficulty awaiting turn”. High scores on an item thus indicated high level of problems in that area. Mean scores of the 9 items in each of the two dimensions were calculated.

### Covariates

In gestational week 17, women were asked about their psychological and psychiatric problems prior to pregnancy (anxiety, depression, or eating disorders), height and pre-pregnancy weight, educational level, and smoking. Three years after delivery, women completed a short version of the Adult ADHD Self-Report Scale^30^, including four items on inattention and two items on impulsivity/hyperactivity from DSM-IV criteria of ADHD. Examples are: “When you have a task that requires a lot of thought, how often do you avoid or delay getting started?” and “How often do you fidget or squirm with your hands or feet when you have to sit down for a long time?” The items were rated on a five-point scale ranging from “never” to “very often”. A mean score was computed from these six items. This short scale is a good predictor of clinically assessed ADHD^30^, and has been used to adjust for maternal ADHD in previous studies of prenatal exposures and child outcomes^21,25^. The Medical Birth Registry of Norway provided information about parity, maternal age, and the child’s year of birth.

Potential multicollinearity between covariates can be expressed as their tolerance (i.e. the proportion of variance in each covariate independent of other covariates)^31^. Tolerance below 0.10 is often considered problematic^31^. All covariates had tolerance above 0.75.

### Statistical analyses

The association between fever at any time during pregnancy and a registry-based ADHD diagnosis was examined in 99,947 children using logistic regression, because of the dichotomous nature of the outcome (ADHD diagnosis in NPR - yes/no). Associations between maternal fever episodes in different trimesters and child ADHD were also examined with logistic regression. Odds ratios (OR) and associated 95% confidence intervals (CI) were calculated. ORs are interpretable as the odds for the outcome among exposed children divided by the odds among unexposed children. For relatively infrequent outcomes (i.e. 10% or less), OR is very similar to relative risk (RR)^32^.

Maternal fever and mother-reported ADHD symptoms at 8 years were examined in linear regression models. Mean scores of the nine inattention symptoms and the nine hyperactivity/impulsivity symptoms were log-transformed due to non-normality, standardized and used as outcomes in separate models. Results were interpretable in terms of units of standard deviation differences in the outcome between exposed and unexposed children (i.e. Cohen’s d).

Then, children of women taking versus not taking acetaminophen for their fever were allocated to different groups, and compared to children of mothers unexposed to fever. This was done with logistic regression for ADHD diagnosis and with linear regression for the mother-reported symptoms, adjusting for covariates as described above. Three dummy-coded variables - no fever, fever without acetaminophen, and fever with acetaminophen - were constructed. The latter two were used as predictors, and the first (no fever) as reference category.

Based on previous literature^15,21,25^, we adjusted for the following potential confounders: maternal parity, history of psychological or psychiatric problems, age and educational level, pre-pregnancy BMI, smoking, and the child’s birth year. A confounder creates a spurious association between two variables by affecting both of them^33^. We therefore only used variables related to both exposure and outcome as potential confounders. Maternal alcohol consumption during pregnancy and child’s sex were considered, but omitted as covariates as they were not related to fever in pregnancy. Analyses also failed to show a clear sex difference in the association between fever in pregnancy and child ADHD diagnosis or symptoms. Boys and girls were therefore analysed together. Maternal ADHD symptoms were included as a covariate in the model even if it was measured three years after the child was born. This was done because of the importance of maternal ADHD as a potential cofounder of associations between prenatal exposures and child ADHD.

Analyses were performed in Mplus version 8^34^. The Full Information Maximum Likelihood estimator was used, which allows using all data from participants with missing information.

Sensitivity analyses: Some children may have received an ADHD diagnosis before 2008 – the first year of person identifiable diagnoses in the NPR. They would not be registered in the NPR if they only had contact with primary care from 2008. To examine potential bias because of this, the association between maternal fever anytime during pregnancy and child ADHD diagnosis was examined with logistic regression, adjusted for the same covariates as above, excluding children born before 2003.

Third-trimester fever was measured in the second and third questionnaires, without information about temperature in the latter. Hence, women with temperatures below 38.5 degrees Celsius may have been included in the fever category in the third trimester, possibly attenuating the association between third-trimester fever and ADHD. To examine this, logistic regression analyses were performed excluding women reporting third-trimester fever only in the third questionnaire. ADHD diagnosis was included as outcome, with fever and the same covariates as above as predictors.

## Results

By the end of the follow-up period (December 2016), 2,941 children (3.0%) had an ADHD diagnosis registered in the NPR. Of those, 92.6% had at least two registrations. At that time, the mean age of the children was 11 years (range 7 to 17).

Characteristics of study participants are presented in Table 1. Using the ADHD Rating Scale, children with an ADHD diagnosis scored on average 2.2 standard deviations higher than children without a registry diagnosis on symptoms of inattention and hyperactivity/impulsivity at age 8 years (p < 0.01).

**Table 1:** Descriptive statistics by maternal fever status.

	Total sample^1^ N = 99,947	Mother did not have fever during pregnancy N = 74,572	Mother had fever during pregnancy N = 9,100
	Mean (SD) or N(%)	Mean (SD) or N(%)	Mean (SD) or N(%)
**Maternal age at delivery**			
24 years or younger	11,175 (11.2%)	7,559 (10.1%)	1,090 (12.0%) **
25–34 years	71,372 (71.4%)	53,961 (72.4%)	6,497 (71.4%)
35 years or older	17,400 (17.4%)	13,052 (17.5%)	1,513 (16.6%)
**Maternal education**			
University/college	60,693(60.7%)	46,842 (62.8%)	5,576 (61.3%) **
High school	26,441 (26.5%)	19,245 (25.8%)	2,382 (26.2%) **
Less than high school	7,658 (7.7%)	4,817 (6.5%)	692 (7.6%)
Missing information	5,155 (5.2%)	3,668 (4.9%)	450 (4.9%)
Maternal mental health problems before pregnancy	9,786 (9.8%)	6,820 (9.1%)	1,101 (12.1%)**
Maternal ADHD symptoms	1.09 (0.58)	1.08 (0.57)	1.14 (0.60)**
Maternal BMI^2^	24.0 (4.3)	24.0 (4.2)	24.4 (4.7)**
Maternal smoking	8,334 (8.5%)	5,463 (7.4%)	839 (9.4%)**
Parity = 0	44,770 (44.8%)	34,958 (46.9%)	3,232 (35.5%)**
Child’s sex = boy	51,151 (51.2%)	38,199 (51.2%)	4,604 (50.6%)
Child’s age 2016 (Range)	11.4 (2.2) (7.4–17.3)	11.4 (2.1) (7.4–16.8)	11.3 (2.2)** (7.5–17.1)
Child registered with ADHD diagnosis	2,941 (3.0%)	2,052 (2.8%)	350 (3.9%) **
ADHD symptoms (inattention) at 8 years^3^	1.55 (0.46)	1.55 (0.45)	1.58 (0.48)**
ADHD symptoms (hyper/imp) at 8 years^3^	1.39 (0.43)	1.39 (0.43)	1.41 (0.46)**

Notes: ^1^All singletons in MoBa with information from the questionnaire in gestational week 17. ^2^BMI when mother became pregnant. ^3^ADHD symptoms are mean scores of nine items on inattention and nine items on hyperactivity/impulsivity, respectively, rated by mother from 1 (“Never/rarely”) to 4 (“very often”). The number of children with information in the 8-year questionnaire was 41,543. **Difference between mothers with versus without fever in pregnancy is statistically significant at p < 0.01.

A total of 9,100 children were exposed to maternal fever during pregnancy (9.1%). Frequencies of fever and acetaminophen use in different trimesters are presented in the last two columns in Table 2.

**Table 2:** Associations between fever in the different trimesters and offspring ADHD diagnoses and symptoms of inattention and hyperactivity/impulsivity.

	ADHD diagnosis^1^ OR		Inattention symptoms^2^ Cohen’s d		Hyperactivity/impulsivity symptoms^2^ Cohen’s d		Number of mothers with fever	Number of mothers using acetaminophen for their fever
	Unadjusted for covariates^3^ 95% C.I.	Adjusted^4^ 95% C.I.	Unadjusted for covariates^3^ 95% C.I.	Adjusted^4^ 95% C.I.	Unadjusted for covariates^3^ 95% C.I.	Adjusted^4^ 95% C.I.		
Any time	1.45*** 1.29–1.62	1.30*** 1.15–1.47	0.07*** 0.03–0.11	0.06** 0.02–0.10	0.05** 0.01–0.09	0.03 –0.01–0.07	9,100	5,075
First trimester	1.36** 1.11–1.68	1.31* 1.06–1.61	0.11** 0.05–0.17	0.09** 0.03–0.15	0.06 0.00–0.12	0.04 −0.02–0.10	3,122	1,174
Second trimester	1.23* 1.04–1.45	1.13 0.95–1.34	0.06* 0.02–0.10	0.05* 0.01–0.09	0.04 0.00–0.08	0.02 −0.02–0.06	5,352	3,526
Third trimester	1.13 0.82–1.56	1.06 0.77–1.46	−0.02 −0.10–0.06	−0.01 −0.09–0.07	0.04 −0.04–0.12	0.03 −0.05–0.11	1,444	725

Notes: ***p < 0.001, **p < 0 0.01, *p < 0.05. ^1^Analyses of fever at any time during pregnancy included all children with information from the questionnaire in gestational week 17 (n = 99,947). Analyses of fever in the different trimesters included children with information from questionnaires in gestational weeks 17 and 30, as well as 6 months after birth (n = 82,264). ^2^Analyses of fever at any time during pregnancy included children born before 2009 with information from the first questionnaire (n = 96,794). Analyses of fever in the different trimesters included children born before 2009 with information from questionnaires completed in gestational weeks 17 and 30, as well as 6 months after birth (n = 79,773). The Full Information Maximum Likelihood estimator was used. Hence, participants who had not returned follow-up questionnaires were also included in the analyses. ^3^Unadjusted estimates: adjusted for child’s birth year, and fever in different trimesters are adjusted for each other. ^4^Analyses were adjusted for the following covariates: maternal age, maternal educational level, parity, maternal pre-pregnancy BMI, maternal pre-pregnancy psychological and psychiatric problems, maternal ADHD symptoms, maternal smoking, and child’s birth year.

**Figure 2:**
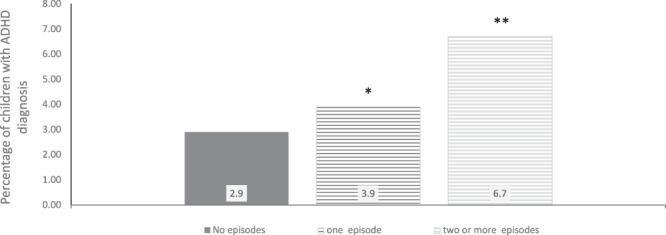
Percentage of children with an ADHD diagnosis by number of maternal fever episodes in the first trimester. Notes: */**Differs from unexposed group at p < 0.05/p < 0.01 in logistic regression analyses adjusted for maternal age, maternal educational level, parity, maternal pre-pregnancy BMI, maternal pre-pregnancy psychological and psychiatric problems, maternal smoking, maternal ADHD symptoms, and child’s birth year. N in analysis = 82,264.

The adjusted OR between fever at any time during pregnancy and offspring ADHD diagnosis was 1.30 (CI = 1. 15–1. 47) (Table 2). First-trimester fever was associated with offspring ADHD after adjusting for covariates and fever in the other trimesters (OR = 1.31, CI = 1.06–1.61). The OR was 2.64 (CI = 1.36–5.14) for 178 children exposed to fever twice or more in the first trimester (see Fig. 2).

Fever at any time in pregnancy was associated with inattention symptoms reported in the 8-year questionnaire (Table 2). The adjusted associations were positive and statistically significant for fever in the first and second trimesters, but this was not seen for fever in the third trimester.

**Figure 3:**
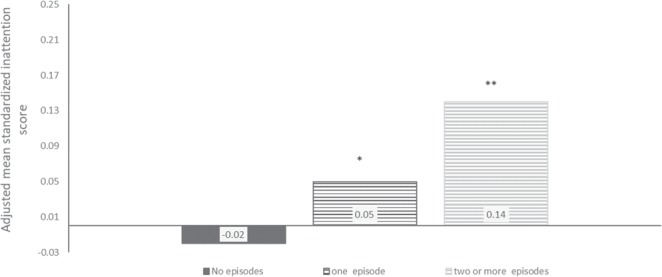
Child inattention scores by number of maternal fever episodes in the first and second trimesters. Notes: The y-axis shows standardized inattention scores (mean of the entire sample = 0, standard deviation = 1). */** Differs from unexposed group at p < 0.05/p < 0.01 in linear regression analyses adjusted for maternal age, maternal educational level, parity, maternal pre-pregnancy BMI, maternal pre-pregnancy psychological and psychiatric problems, maternal smoking, maternal ADHD symptoms, and child’s birth year. The Full Information Maximum Likelihood estimator was used, allowing inclusion of participants who had not returned the 8-year questionnaire. Those who were born before 2009 were not old enough to have received their 8-year questionnaire and were thus not included. N in analyses = 79,773.

Children of 1,631 mothers exposed to fever twice or more during the two first trimesters had the highest inattention scores (Cohen’s d = 0.14, CI = 0.06–0.22). I.e., exposed children scored 0.14 standard deviations higher than unexposed children (see Fig. 3).

Maternal fever at any time during pregnancy was not associated with child hyperactivity/impulsivity symptoms at age 8 after adjusting for covariates. (Table 2).

**Figure 4:**
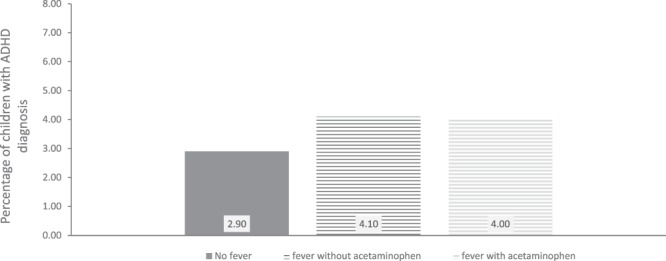
Percentage of children with an ADHD diagnosis by maternal fever and acetaminophen use in first trimester. Note: N = 82,264.

**Figure 5:**
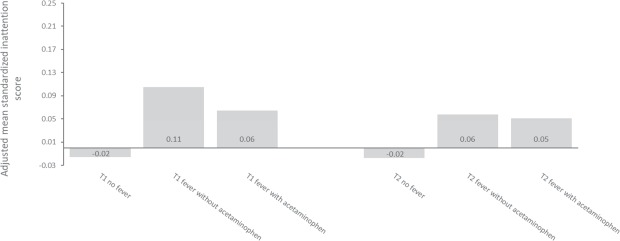
Child inattention scores by maternal fever and acetaminophen use in first and second trimester. Notes: The y-axis shows standardized inattention scores (mean of the entire sample = 0, standard deviation = 1). Adjusted for maternal age, maternal educational level, parity, maternal pre-pregnancy BMI, maternal pre-pregnancy psychological and psychiatric problems, maternal smoking, maternal ADHD symptoms, and child’s birth year. The Full Information Maximum Likelihood estimator was used, allowing inclusion of participants who had not returned the 8-year questionnaire. Those who were born before 2009 were not old enough to have received their 8-year questionnaire and were thus not included. N in analyses = 79,773.

Children of mothers with fever had similar odds of receiving an ADHD diagnosis whether or not the mother had taken acetaminophen (OR = 1.35, CI = 0.96–1.90 with acetaminophen, and OR = 1.32, CI = 1.01–1.71 without acetaminophen) (see Fig. 4). Children of women who had taken acetaminophen for their fever in the first or second trimester, had similar inattention scores as children of mothers who had not taken acetaminophen for their fever. First trimester fever with acetaminophen: Cohen’s d = 0.08, CI = −0.02–0.18; without acetaminophen: Cohen’s d = 0.10, CI = 0.02–0.18. Fever in second trimester with acetaminophen: Cohen’s d = 0.04, CI = −0.02–0.10; without acetaminophen: Cohen’s d = 0.06, CI = −0.02–0.14, (See Fig. 5).

### Sensitivity analyses

The adjusted association between maternal fever anytime during pregnancy and child ADHD diagnosis was OR = 1.33 (p < 0.001) when excluding children born before 2003.

Excluding women reporting third-trimester fever only in the third questionnaire (without temperature information), did not increase the association between third-trimester fever and ADHD (OR = 0.80, p > 0.05).

## Discussion

Children exposed to maternal fever during pregnancy had increased risk of receiving an ADHD diagnosis and of elevated inattention symptoms at age 8 years. Increased risk of ADHD diagnosis was primarily associated with first-trimester fever. Inattention symptoms were associated with fever in the first, and, to some extent, the second trimester. There was no association between fever and hyperactivity/impulsivity after adjusting for covariates.

Most research on maternal fever during pregnancy has focused on congenital malformations (e.g., neural tube defects, congenital heart defects, oral clefts^7^). Associations with autism, cerebral palsy, developmental delay, decreased academic performance, psychosis, lack of task persistence and negative emotionality have also been shown^3,6–8^. Our results extend these observations by demonstrating an increased risk of ADHD diagnosis.

One previous study of prenatal fever and ADHD diagnosis found the association to be restricted to fever in gestational weeks 9 to 12^15^. Our results provide support for the highest risk being associated with exposure in early pregnancy, but the findings also suggest that inattention is associated with fever in a wider time period. The current study showed for the first time that repeated fever episodes in the beginning of pregnancy were associated with increased risk for both ADHD diagnosis and inattention symptoms. Children exposed twice or more, had more than two and a half times higher odds of getting an ADHD diagnosis compared to unexposed children, and they had the highest inattention symptoms.

Timing of prenatal exposures may influence the risk, with different disorders potentially resulting from variation in exposure across pregnancy periods^35^. Such trimester differences could reflect influences on various phases of brain development. The brain starts developing early in the first trimester, with neuron production beginning in the last part of the first trimester^36^. During the second trimester, the majority of neurons will have migrated to their final locations^3^. A recent study from MoBa showed that second-trimester fever was particularly associated with risk of autism spectrum disorders among offspring^6^. Our results suggest that both ADHD diagnosis and symptoms of inattention may be linked to maternal fever in early pregnancy. However, timing effects require verification over multiple studies.

Several maternal infections and autoimmune diseases during pregnancy are associated with neurodevelopmental disorders in the offspring, suggesting that factors common to these conditions may contribute to the increased risk^35,37^. Fever has been proposed as such a common factor^4,35^, and our study adds to previous knowledge by examining the association between maternal fever and child ADHD directly, taking timing and repeated exposures into account. Hyperthermia during pregnancy is linked to several adverse outcomes in offspring, including growth retardation and developmental defects in both animal models^5,14^ and humans^5^. Hence, elevated temperature alone may exert teratogenic effects on the fetus, and may have detrimental effects on central nervous system development^5,35^. If hyperthermia is the mechanism linking maternal fever to offspring ADHD, the current study’s focus on higher reported levels of fever may lead to more accurate results.

An alternative explanation of the association between fever and child ADHD is that fever is a marker of another underlying process, for instance specific infections or immune disturbances, that contribute to the increased risk for ADHD. Fever reflects levels of pro-inflammatory cytokines as well as other molecules^38^, thus, maternal cytokine levels may cross the placenta to affect fetal brain development via mechanisms other than hyperthermia^35^.

The overall effect sizes were small in the current study. They are comparable to associations between prenatal exposures and child outcomes from other prospective cohort studies, which typically provide weaker estimates than those reported from case-control studies^7^. One explanation for this may be decreased risk of differential recall bias when data are collected prospectively^7,39^. It has been proposed that multiple environmental factors are involved in the development of ADHD, each with small effects^40,41^. If this model is correct, for each individual environmental factor studied, we would not expect more than weak associations. This has been seen as a parallel to the thousands of genetic variants that together affect ADHD, each variant with extremely weak associations to the disorder^2,40,41^.

The current associations between fever (anytime in pregnancy or in the first trimester) and ADHD diagnosis are similar to associations between maternal fever in pregnancy and neurodevelopmental disorders or related problems, as reported in previous prospective cohort studies (e.g. hazard ratio (HR) = 1.4 for autism, HR = 1.5 for cerebral palsy, relative risk (RR) = 1.1 for psychosis-like experiences, OR = 1.3 for academic problems, and OR = 1.3 for lack of task persistence^7^). The association we found between multiple fever episodes in the first trimester and ADHD diagnosis was somewhat higher than these previously reported estimates, and lower than a reported association between maternal fever and schizophrenia (OR = 3.7)^7^.

Effect sizes in our study are in line with previously reported associations between several prenatal exposures and different child outcomes from prospective cohort studies, such as maternal fever and neural tube defects (OR = 1.8)^7^, maternal anorexia nervosa and child being small for gestational age (RR = 1.5)^42^, acetaminophen use and ADHD (RR = 1.3)^43^, maternal fever and birth defects (RR = 2.3)^7^, and smoking and low birth weight (OR = 1.9)^44^. Regarding mother-reported symptoms in our study, associations with fever were comparable to several previous studies of different prenatal exposures and continuous child outcomes (e.g. maternal alcohol use and child ADHD symptoms (Beta = 0.05)^45^, and maternal smoking during pregnancy and internalizing symptoms in children (Beta = 0.11 and 0.06 – interpretable as Cohen’s d in that study^46^)). The association between exposure to multiple fever episodes in the beginning of pregnancy and inattention symptoms in our study was also similar to a previously reported association between binge alcohol exposure in pregnancy and child cognition reported in a meta-analysis (Cohen’s d = 0.13)^47^.

The implications of reported effect sizes such as ORs, RRs and HRs, depend on the absolute risk of the disorder and the exposure, as well as on the duration and severity of the disorder. Both maternal fever in pregnancy and ADHD in children are common phenomena^7,48^. Hence, even a weak association between maternal fever and child ADHD may be important at a population level. This is emphasized by ADHD’s low age of onset, endurance into adulthood, and the fact that the disorder is associated with several adverse outcomes (e.g. academic problems, substance misuse, incarcerations and mortality^2^). Nevertheless, the current results show that each individual child exposed to fever in pregnancy, will most likely not develop ADHD or inattention problems.

ADHD is highly heritable, and the observed association between maternal fever and child ADHD could be confounded by genetic factors. We adjusted for maternal ADHD symptoms in the current study, but the association could still be influenced by unmeasured common genetic or environmental factors that increase risk for both ADHD and fever. The current study included fever episodes from several trimesters in the same model. Stable confounding factors such as maternal general health, reporting bias, socioeconomic status, or genetic risk might be expected to affect fever in all three trimesters, and are less likely as explanations for trimester-specific associations with child ADHD. However, we cannot rule out trimester-specific confounding.

Acetaminophen use in pregnancy has previously been shown to be associated with ADHD^20,21^. In a recent study from MoBa, long-term use of acetaminophen during pregnancy was associated with increased risk of offspring ADHD. However, short-term use was associated with reduced risk. These findings are concordant with those obtained in a study on autism risk in the MoBa where use of acetaminophen was associated with reduced risk in mothers with fever during pregnancy^6^. Our results showed that risk of ADHD diagnosis and level of inattention symptoms were similar whether the mother had taken acetaminophen for their fever or not. This suggests that the association between fever in pregnancy and child ADHD is not due to detrimental effects of acetaminophen use among women with fever. Further, the observation that use of acetaminophen is insufficient to mitigate the fever-associated risk is consistent with a model wherein a larger set of innate immune responses contribute to outcomes of ADHD and inattention. One way to dissect the contributions of the innate immune system using questionnaire data would be to examine the impact of Non-Steroidal Anti-Inflammatory Drugs (e.g. ibuprofen). However, numbers are small, as pregnant women are often advised against these drugs during pregnancy. Very few women in the current study had used antipyretic medication other than acetaminophen.

The current study has not identified microbial causes of fever and immune responses. Further studies should examine what prenatal infections and inflammatory responses may increase the risk of negative outcomes in the offspring^6^.

Strengths of the study include its large sample size and prospective data collections. However, several limitations warrant consideration. Only about 40% of the invited women consented to participation, potentially reducing the generalizability of our findings. Women younger than 25 years, with low educational level and smokers were under-represented^44^. However, ADHD prevalence, psychosocial adversity and child global functioning were found to be similar in MoBa and in the general Norwegian population^49^. Also, associations between variables tend to be more robust against selective non-response than estimates of means and frequencies^44,50^.

Some participants responding to the first questionnaire did not return later questionnaires. An advantage of the current study was inclusion of registry-based diagnostic outcome for all children, independent of questionnaire responses. For trimester-specific analyzes, only women who had responded to all three fever-questionnaires were included, potentially introducing bias. However, reports on fever in the first questionnaire revealed only minimal differences between those who did and did not return the later questionnaires at gestational week 30 and 6 months after birth. Among the former, 3.1% reported fever, 0.2% had two or more episodes, and 1.2% used acetaminophen for their fever in the first trimester. Among the latter, the corresponding numbers were 3.3%, 0.2%, and 1.1%. In addition, a Danish study found small effects of non-response at follow-up in a similar birth cohort^51^.

A strength in the current study is the repeated measures of fever during pregnancy. Nevertheless, self-reported fever may have low reliability and thus attenuate association estimates. Some studies may indicate that false negatives are more common than false positives regarding self-reported fever^52,53^, meaning that under-reporting may be the biggest problem. Because the group of women without fever is very large in the current study, some false negatives is unlikely to affect results heavily. We were not able to detect multiple fever episodes within each four-week period in the first trimester. Hence, some women actually experiencing multiple fever episodes (e.g. two episodes in gestational weeks 0–4), may have been coded as having had only one fever episode. These women did thus not contribute to the estimate of associations between multiple fever episodes and child outcomes.

Self-reported fever may also be subject to systematic biases, if over- or underestimation of temperature is related to variables associated with child ADHD. For example, the current associations may be inflated or deflated if women with ADHD for some reason systematically over- or under-report fever more often than women without ADHD.

The proportion of women reporting fever during pregnancy in the current study was lower than in the Danish birth cohort^15^ and The National Birth Defects Prevention study^54^. This may be due to under-reporting of fever in the current study, or due to our definition of fever as temperature above 38.5 degrees Celsius. In the Danish birth cohort, most of the fever cases were classified as low intensity (low temperature and/or short duration).

Information about acetaminophen use was only a yes/no variable for each fever period, without information about duration or dosage. We cannot rule out that results would have been different with more detailed information about this.

We did not have information about women’s substance use or psychotic episodes before or during pregnancy. This may have biased results.

Some children may have undiagnosed ADHD. This may be particularly relevant for the youngest children, as ADHD prevalence within the NPR increases from age 5 to age 11^55^. At end of follow-up, only 3.2% of the children in our sample were 7 years old, and half of the children were 11 years old or more. Age differences in the likelihood of having received a diagnosis was accounted for by adjusting analyses for child’s birth year.

## Conclusion

The results indicate that maternal fever in early pregnancy may be a risk factor for ADHD, and particularly for inattention problems. Children exposed twice or more in the first trimester, had more than doubled odds of receiving an ADHD diagnosis compared to unexposed children. This risk was neither mitigated nor increased by use of acetaminophen.

Strategies that minimize the risk of maternal infection, the most common cause of fever, may contribute to reducing ADHD and inattention problems in children. Future studies should investigate the mechanisms by which maternal fever might create risk for adverse neurodevelopmental outcomes such as ADHD, as well as scrutinize the efficacy of different antipyretics in mitigating such risk.

## Data Availability

Data from the Norwegian Mother and Child Cohort Study and registers used in this study are managed by the national health register holders in Norway and can be made available to researchers, provided necessary approval from the Regional Ethics Committee in Norway and from the data owners. The Norwegian Institute of Public Health has a general contact point for data access at the mail-address: datatilgang@fhi.no.

## References

[1] Silva D, Colvin L, Hagemann E, Bower C (2014). Environmental risk factors by gender associated with attention-deficit/hyperactivity disorder. Pediatrics.

[2] Thapar A, Cooper M (2016). Attention deficit hyperactivity disorder. The Lancet.

[3] Dombrowski SC, Martin RP, Huttunen MO (2003). Association between maternal fever and psychological/behavior outcomes: A hypothesis. Birth Defects Res A.

[4] Martin, R. P. & Dombrowski, S. C. *Prenatal exposures: Psychological and educational consequences for children*. (Springer, 2008).

[5] Edwards MJ (2006). Review: Hyperthermia and fever during pregnancy. Birth Defects Res A Clin Mol Teratol.

[6] Hornig M (2017). Prenatal fever and autism risk. Mol Psychiatry.

[7] Dreier JW, Andersen AM, Berg-Beckhoff G (2014). Systematic review and meta-analyses: fever in pregnancy and health impacts in the offspring. Pediatrics.

[8] Zerbo O (2013). Is maternal influenza or fever during pregnancy associated with autism or developmental delays? Results from the CHARGE (CHildhood Autism Risks from Genetics and Environment) study. J Autism Dev Disord.

[9] Milunsky A (1992). Maternal heat exposure and neural tube defects. JAMA.

[10] Chambers CD (2006). Risks of hyperthermia associated with hot tub or spa use by pregnant women. Birth Defects Res A.

[11] Edwards MJ (1986). Hyperthermia as a Teratogen: A Review of Experimental Studies and Their CIinical Significance. Teratogenesis, Carcinogenesis, and Mutagenesis.

[12] Hinoue A, Fushiki S, Nishimura Y, Shiota K (2001). In utero exposure to brief hyperthermia interferes with the production and migration of neocortical neurons and induces apoptotic neuronal death in the fetal mouse brain. Brain Res Dev Brain Res.

[13] Holmes LB (2011). Human teratogens: update 2010. Birth Defects Res A Clin Mol Teratol.

[14] Edwards MJ, Saunders RD, Shiota K (2003). Effects of heat on embryos and foetuses. Int J Hyperther.

[15] Dreier JW (2016). Fever and infections in pregnancy and risk of attention deficit/hyperactivity disorder in the offspring. The Journal of Child Psychology and Psychiatry.

[16] Milich R, Balentine AC, Lynam DR (2001). ADHD combined type and ADHD predominantly inattentive type are distinct and unrelated disorders. Clinical Psychology-Science and Practice.

[17] Ask H (2018). Association of Gestational Age at Birth With Symptoms of Attention-Deficit/Hyperactivity Disorder in Children. JAMA pediatrics.

[18] Kuntsi J (2014). The separation of ADHD inattention and hyperactivity-impulsivity symptoms: pathways from genetic effects to cognitive impairments and symptoms. J Abnorm Child Psychol.

[19] Brandlistuen RE, Ystrom E, Nulman I, Koren G, Nordeng H (2013). Prenatal paracetamol exposure and child neurodevelopment: a sibling-controlled cohort study. Int J Epidemiol.

[20] Liew Z, Ritz B, Rebordosa C, Lee PC, Olsen J (2014). Acetaminophen use during pregnancy, behavioral problems, and hyperkinetic disorders. JAMA pediatrics.

[21] Ystrom E (2017). Prenatal expopsure to Acetaminophen and risk of ADHD. Pediatrics.

[22] Magnus P (2006). Cohort profile: The Norwegian Mother and Child Cohort Study (MoBa). International Journal of Epidemiology.

[23] Magnus P (2016). Cohort Profile Update: The Norwegian Mother and Child Cohort Study (MoBa). Int J Epidemiol.

[24] Schafer JL, Graham JW (2002). Missing data: Our view of the state of the art. Psychological Methods.

[25] Gustavson Kristin, Ystrom Eivind, Stoltenberg Camilla, Susser Ezra, Surén Pål, Magnus Per, Knudsen Gun Peggy, Smith George Davey, Langley Kate, Rutter Michael, Aase Heidi, Reichborn-Kjennerud Ted (2017). Smoking in Pregnancy and Child ADHD. Pediatrics.

[26] Zhu JL (2014). Parental smoking during pregnancy and ADHD in children: the Danish national birth cohort. Pediatrics.

[27] *The ICD-10 classification of mental and behavioural disorders: Clinical descriptions and diagnostic guidelines*, (World Health Organization, 1992).

[28] Silva RR (2005). A rating scale for disruptive behavior disorders, based on the DSM-IV item pool. Psychiatr Q.

[29] American Psychiatric Association. *Diagnostic and Statistical Manual of Mental Disorders. Text Revision*. Foruth edn, (APA, 2000).

[30] Kessler RC (2005). The World Health Organization Adult ADHD Self-Report Scale (ASRS): a short screening scale for use in the general population. Psychol Med.

[31] Cohen, J., Cohen, P., West, S. G. & Aiken, L. S. *Applied multiple regression/correlation analysis for the behavioral sciences*. Third edn, (Routledge, 2003).

[32] Zhang J, Yu KF (1998). What’s the relative risk? A method of correcting the odds ratio in cohort studies of common outcomes. JAMA.

[33] Greenland S, Pearl J, Robins JM (1999). Causal diagrams for epidemiologic research. Epidemiology.

[34] Muthén, L. K. & Muthén, B. O. *Mplus User’s Guide*. 8th edn, (Muthén & Muthén, 1998–2017).

[35] Flinkkila E, Keski-Rahkonen A, Marttunen M, Raevuori A (2016). Prenatal Inflammation, Infections and Mental Disorders. Psychopathology.

[36] Stiles J, Jernigan TL (2010). The basics of brain development. Neuropsychol Rev.

[37] Instanes JT (2017). Attention-Deficit/Hyperactivity Disorder in Offspring of Mothers With Inflammatory and Immune System Diseases. Biol Psychiatry.

[38] Romanovsky AA (2005). Fever and hypothermia in systemic inflammation: recent discoveries and revisions. Front Biosci.

[39] Hashmi SS (2015). Studying the effects of maternal febrile exposures on neurological outcomes in offspring: conceptual and methodological issues. Dev Med Child Neurol.

[40] Sonuga-Barke, E. In *ADHD and hyperkinetic disorder* (eds Banaschewski, T. & Zuddas, A.) Ch. 3, (Oxford university press, 2015).

[41] Taylor, E. & Sonuga-Barke, E. In *Rutter’s child and adolescent psychiatry* (eds Rutter, M. *et al*.) Ch. 34, (Blackwell, 2008).

[42] Watson HJ (2017). Maternal eating disorders and perinatal outcomes: A three-generation study in the Norwegian Mother and Child Cohort Study. J Abnorm Psychol.

[43] Masarwa R (2018). Prenatal Exposure to Acetaminophen and Risk for Attention Deficit Hyperactivity Disorder and Autistic Spectrum Disorder: A Systematic Review, Meta-Analysis, and Meta-Regression Analysis of Cohort Studies. Am J Epidemiol.

[44] Nilsen, R. M. *et al*. Self-selection and bias in a large prospective pregnancy cohort in Norway. *Paediatric and Perinatal Epidemiology* (2009). 10.1111/j.1365-3016.2009.01062.x 19840297

[45] Eilertsen EM (2017). Maternal alcohol use during pregnancy and offspring attention-deficit hyperactivity disorder (ADHD): a prospective sibling control study. Int J Epidemiol.

[46] Moylan S (2015). The impact of maternal smoking during pregnancy on depressive and anxiety behaviors in children: the Norwegian Mother and Child Cohort Study. BMC medicine.

[47] Flak AL (2014). The association of mild, moderate, and binge prenatal alcohol exposure and child neuropsychological outcomes: a meta-analysis. Alcohol Clin Exp Res.

[48] Polanczyk GV, Willcutt EG, Salum GA, Kieling C, Rohde LA (2014). ADHD prevalence estimates across three decades: an updated systematic review and meta-regression analysis. Int J Epidemiol.

[49] Oerbeck B (2017). ADHD, comorbid disorders and psychosocial functioning: How representative is a child cohort study? Findings from a national patient registry. BMC Psychiatry.

[50] Gustavson, K., von Soest, T., Karevold, E. & Roysamb, E. Attrition and generalizability in longitudinal studies: findings from a 15-year population-based study and a Monte Carlo simulation study. *BMC Public Health* **12** (2012). 10.1186/1471-2458-12-918 PMC3503744 23107281

[51] Greene N, Greenland S, Olsen J, Nohr EA (2011). Estimating bias from loss to follow-up in the Danish National Birth Cohort. Epidemiology.

[52] Nguyen AV (2010). Comparison of 3 infrared thermal detection systems and self-report for mass fever screening. Emerg Infect Dis.

[53] Barbara AM, Loeb M, Dolovich L, Brazil K, Russell M (2012). Agreement between self-report and medical records on signs and symptoms of respiratory illness. Prim Care Resp J.

[54] Collier SA, Rasmussen SA, Feldkamp ML, Honein MA (2009). & National Birth Defects Prevention, S. Prevalence of self-reported infection during pregnancy among control mothers in the National Birth Defects Prevention Study. Birth Defects Res A Clin Mol Teratol.

[55] Suren P (2012). Autism spectrum disorder, ADHD, epilepsy, and cerebral palsy in Norwegian children. Pediatrics.

